# Correction: A revised mechanism for how *Plasmodium falciparum* recruits and exports proteins into its erythrocytic host cell

**DOI:** 10.1371/journal.ppat.1010757

**Published:** 2022-08-05

**Authors:** The PLOS Pathogens Staff

There is an error in the article XML that causes the PubMed citation to be indexed incorrectly. The publisher apologizes for the error.
