# Supplementary figures and images for: A revised mechanism for how Plasmodium falciparum recruits and exports proteins into its erythrocytic host cell
Source: PLoS Pathog. 2022 Feb 22;18(2):e1009977. doi: 10.1371/journal.ppat.1009977 (PMC8896661; doi:10.1371/journal.ppat.1009977)

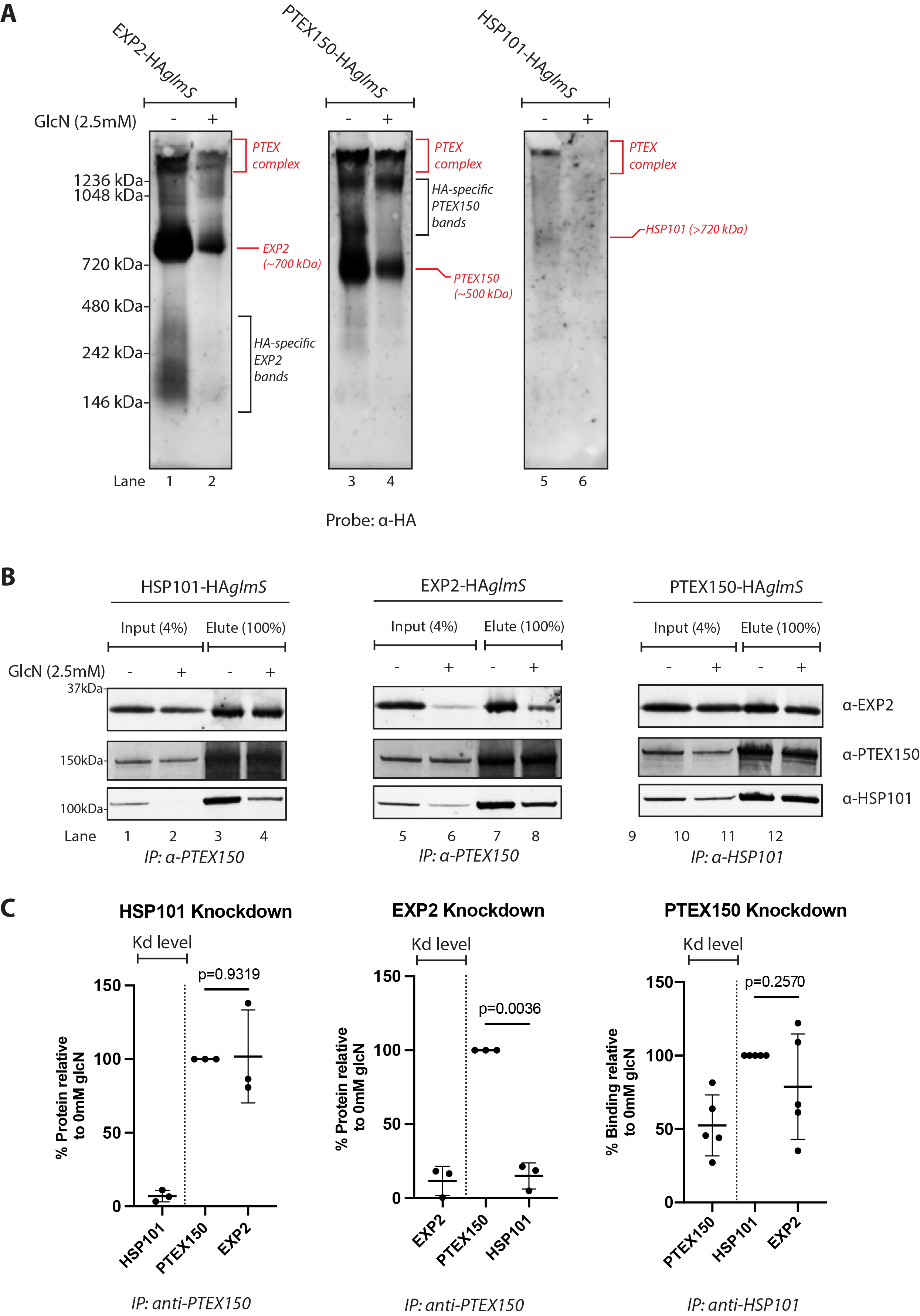

Supplement: S1 Fig — (A) BN-PAGE analysis of EXP2, PTEX150, and HSP101-HAglmS parasite lines following glucosamine treatment. Saponin-lysed pellets were lysed with 1% digitonin, separated on 4–12% NativePAGE gel, and analysed via western blotting using monoclonal anti-HA antibody. The 1236 kDa PTEX bands and the major oligomeric species of EXP2, PTEX150, and HSP101 seen with PTEX-specific antibodies were also recognised by the anti-HA antibody (red text; see Fig 2A, 2B, and 2C). Additional HA-specific bands were also observed in the case of EXP2 and PTEX150-HAglmS (black text). In the case of HSP101-HAglmS, only the 1236 kDa and a faint >720 kDa band were observed (black text), indicating that the 1048 kDa and the 200 kDa bands found with anti-HSP101 antibody (Fig 2A) were likely non-specific. (B) Representative western blots of PTEX co-immunoprecipitated eluates to establish which PTEX components could still form subcomplexes when another component was knocked down (n = 3 independent biological replicates). Parasites expressing HA-glmS tagged PTEX core components were treated +/- 2.5 mM glucosamine (GlcN) and immunoprecipitation (IP) was performed using either anti-PTEX150 antibodies (for HSP101 and EXP2 knockdown) or anti-HSP101 antibodies (for PTEX150 knockdown). (C) Quantification of PTEX band intensity represented in (A) where the amounts of PTEX proteins were normalised to the immunoprecipitated protein. Knockdown (Kd) of HSP101 did not appear to block the interaction between PTEX150 and EXP2 (p = 0.9319, n = 3). Knockdown of EXP2, however, disrupts PTEX150’s interaction with HSP101 (p = 0.0036, n = 3). The knockdown of PTEX150 caused disruption in HSP101’s interaction with EXP2 although this was not statistically significant (p = 0.2570, n = 5) presumably due to residual PTEX150 (approximately 40%) still present in the sample. Plotted data represents the mean ±SD. Statistical significances were measured using an unpaired t-test with Welch’s correction. p-values are indicated on t [file ppat.1009977.s001.tif]

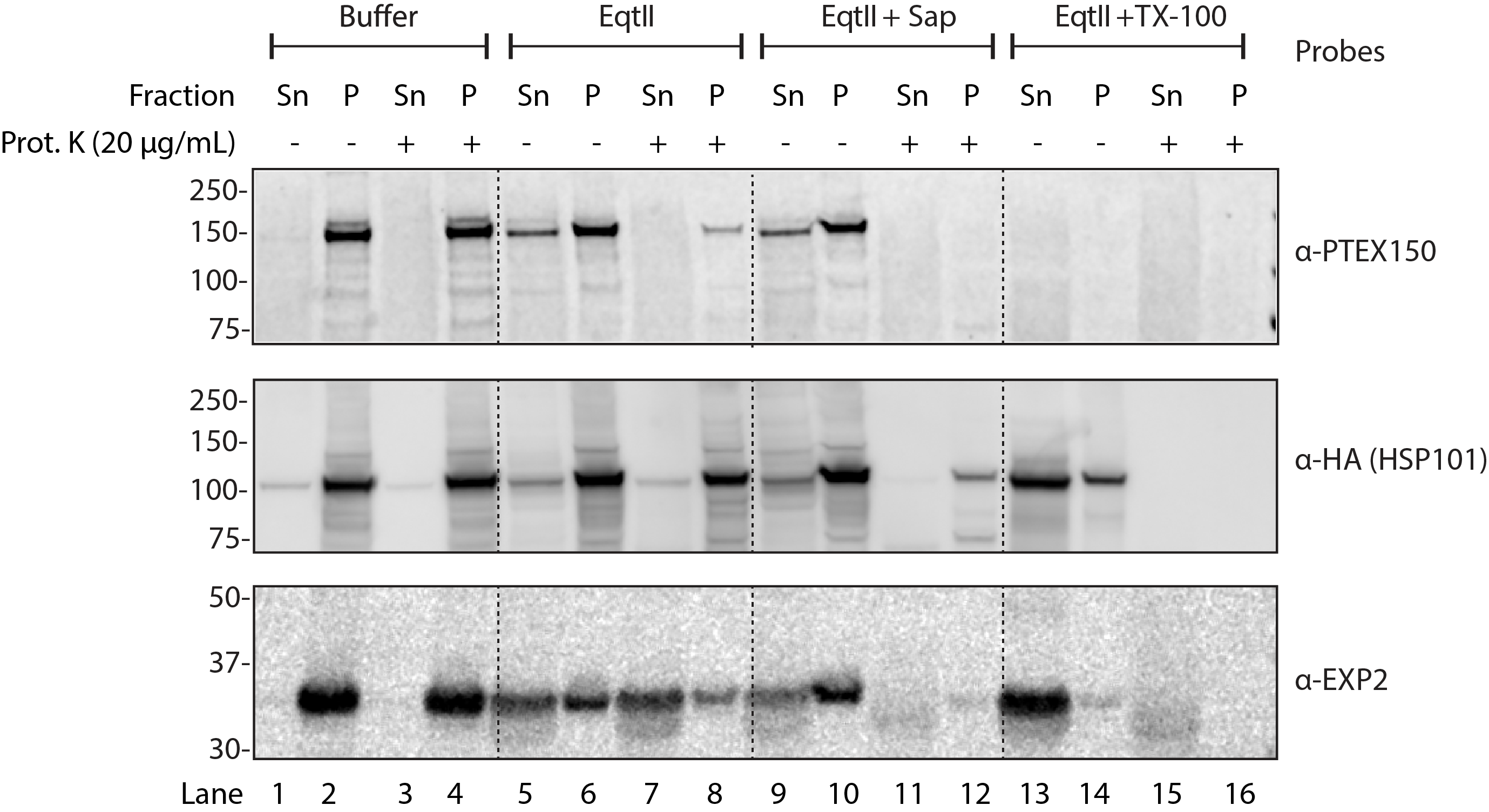

Supplement: S2 Fig — Western blots of trophozoite stage parasites permeabilised with equinatoxin II (EqtII), in combination with 0.03% saponin (sap) or 0.25% Triton X-100 (TX-100) and digested with 20 μg/mL of proteinase K. Buffer only control shows that PTEX150 was present in the starting parasite material. In the absence of protease inhibitor, PTEX150 was completely degraded following permeabilisation with TX-100 even in the absence of proteinase K. The assay was repeated twice. Sn, Supernatant. P, Pellet. (TIF) [file ppat.1009977.s002.tif]

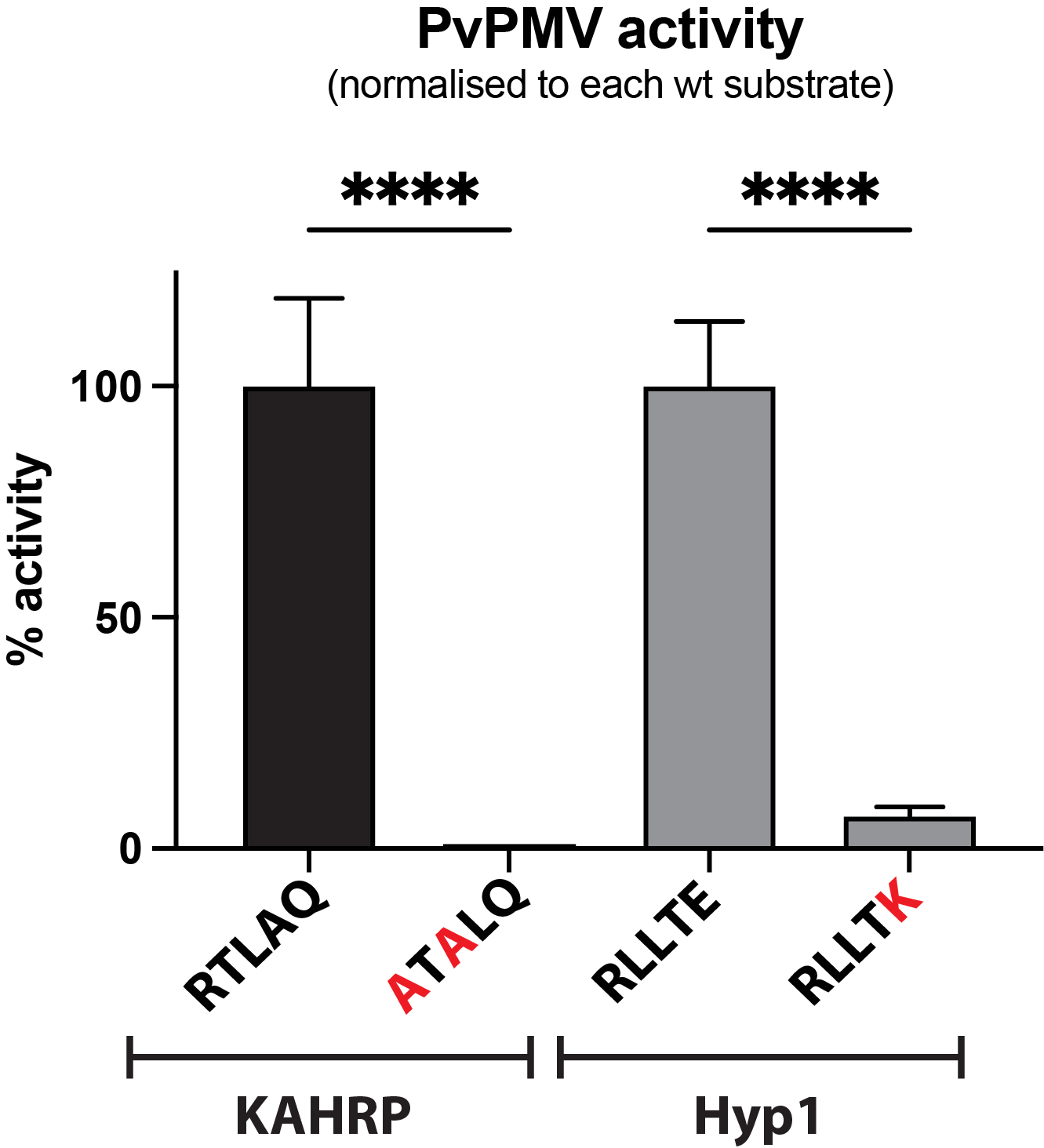

Supplement: S3 Fig — 5 μM fluorogenic peptides were incubated with 2 nM recombinant P. vivax plasmepsin V and assayed at 20°C. Fluorescence data was normalised to the WT substrates (n = 3, error bars = SD) and indicated that the cleavage of P5 Lys Hyp1 peptide (RLLTK) was nearly inhibited to the same level as the double P1 and P3 KAHRP mutant peptide (RTLAQ to ATALQ). Statistical significance was determined using ordinary one-way ANOVA. ****, p-value<0.0001, ***, p-value<0.001. (TIF) [file ppat.1009977.s003.tif]

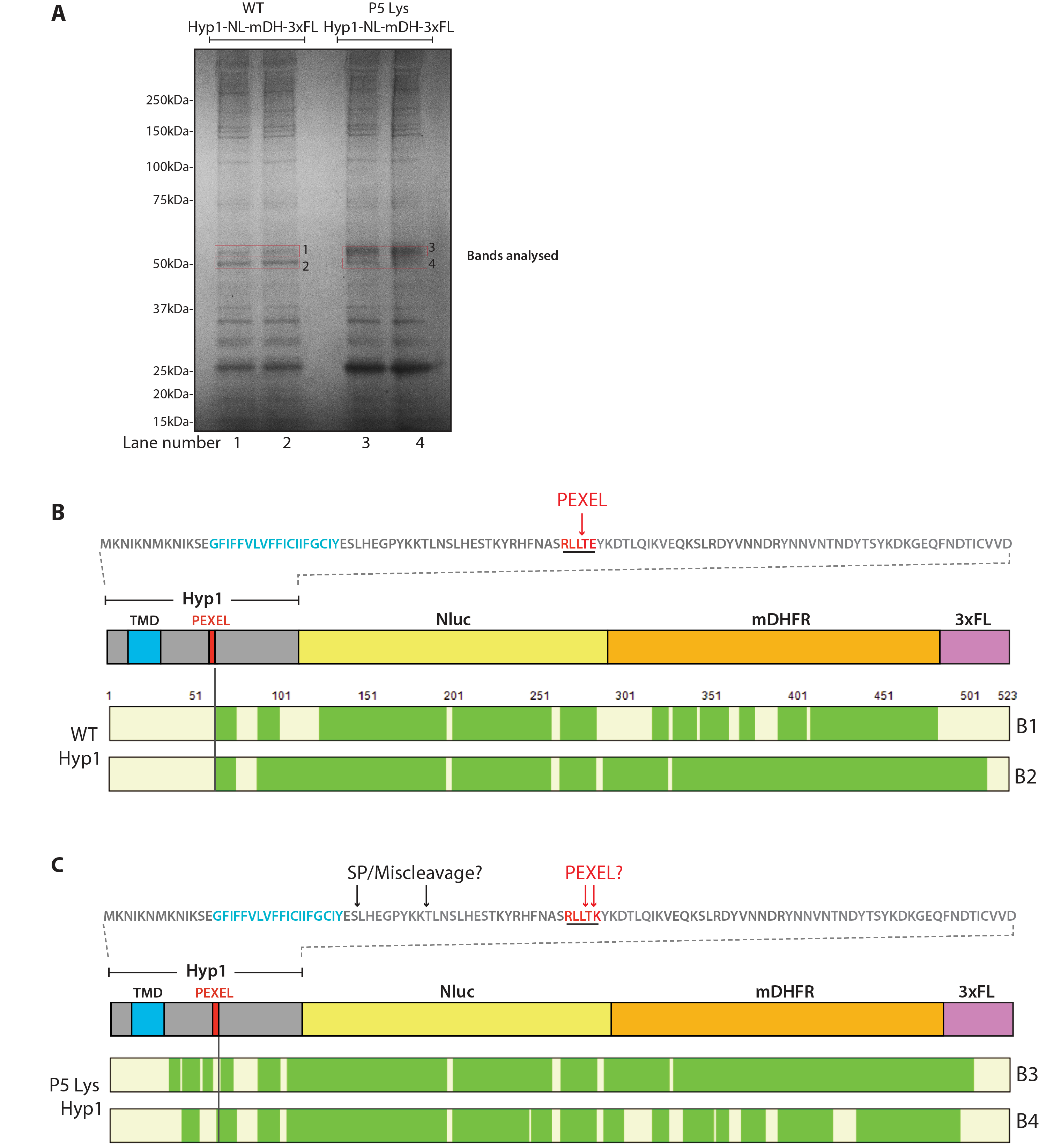

Supplement: S4 Fig — (A) Proteins immunoprecipitated using anti-FLAG IgG beads from parasites expressing WT or P5 Lys Hyp1-Nluc-mDHFR-3xFL reporter proteins were fractionated by SDS-PAGE. Protein bands were visualised with Coomassie stain and protein bands corresponding to the molecular weight of the PEXEL-cleaved WT Hyp1 (band 2) and the miscleaved P5 Lys (band 3) were excised (red boxes). The matching region of the gel for WT (band 1) and P5 Lys (band 4) were also excised and subjected to the same analysis. The protein bands were digested with trypsin or GluC and subjected to mass spectrometry to identify peptide fragments. (B) The amino acid sequence of WT Hyp1 region of Hyp1-Nluc-mDHFR-3xFL protein showing PEXEL motif (underlined, red), peptide cleavage site (arrow), transmembrane domain (blue). Below this is a diagram of the full-length reporter protein with peptide coverage of protein bands 1 and 2 (B1 and B2) indicated in green. (C) Peptide coverage of P5 Lys Hyp1-Nluc-mDHFR-3xFL reporter protein bands 3 and 4 (B3 and B4) as described for (B). Peptides identified between the transmembrane domain and PEXEL motif indicate the mutant protein is processed upstream of the PEXEL motif. (TIF) [file ppat.1009977.s004.tif]

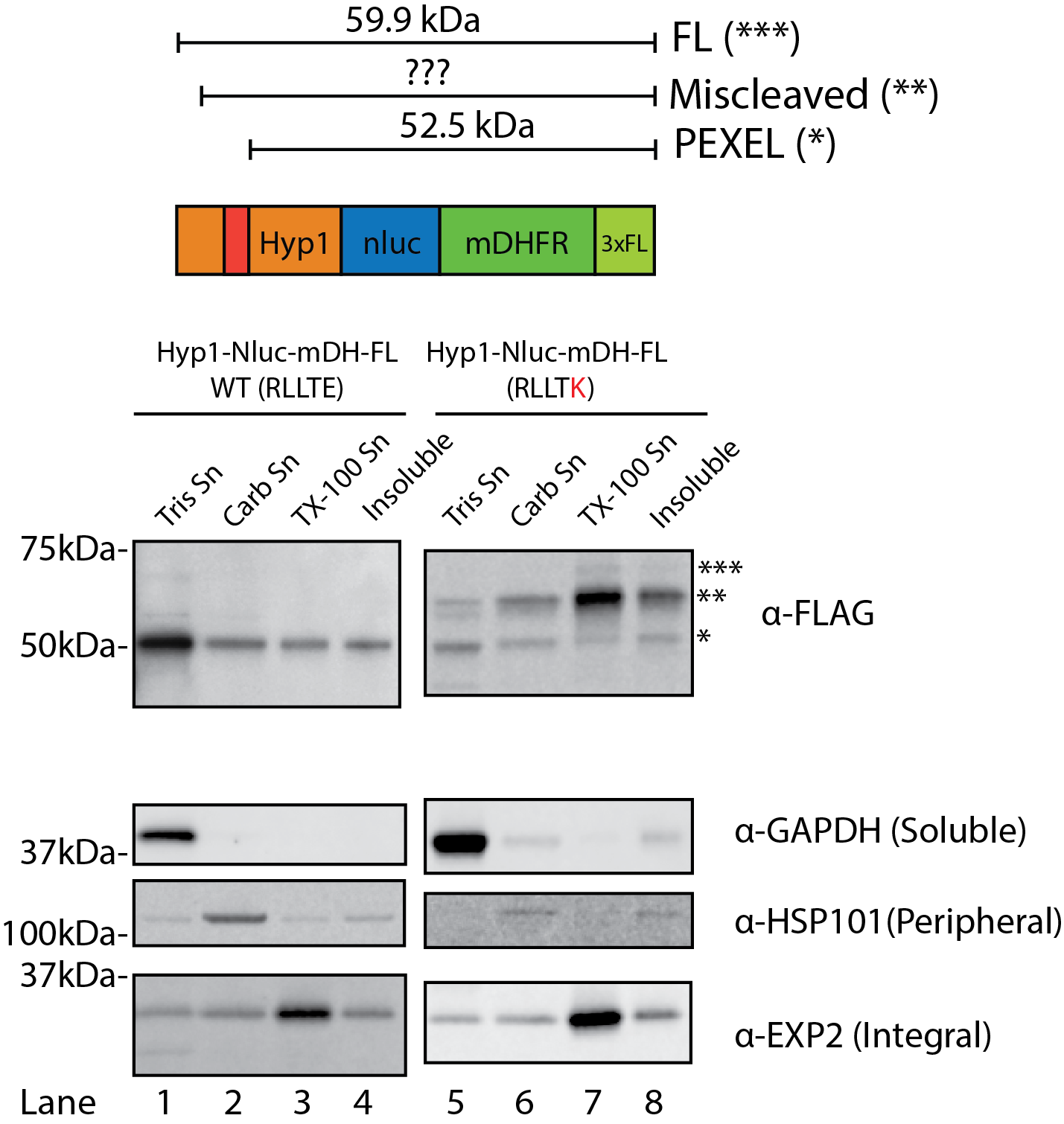

Supplement: S5 Fig — Western blot analysis of infected RBCs (mid-stage trophozoites) sequentially extracted with 5 mM Tris-Cl pH 8.0 (Tris Sn), 0.1 M Na2CO3 pH 11.3 (Carb Sn), and 1% Triton X-100 buffer (TX-100 Sn) to partition proteins based on their association with the cellular membrane. Insoluble fraction represents the final pellet obtained after Triton X-100 extraction. GAPDH, HSP101, and EXP2 were used as a control for the release of soluble, peripheral, and integral protein, respectively. The assay was reproducible in two independent experiments. FL, predicted molecular weight for full-length protein; PEXEL, predicted molecular weight for PEXEL-cleaved protein. Sn, Supernatant. (TIF) [file ppat.1009977.s005.tif]

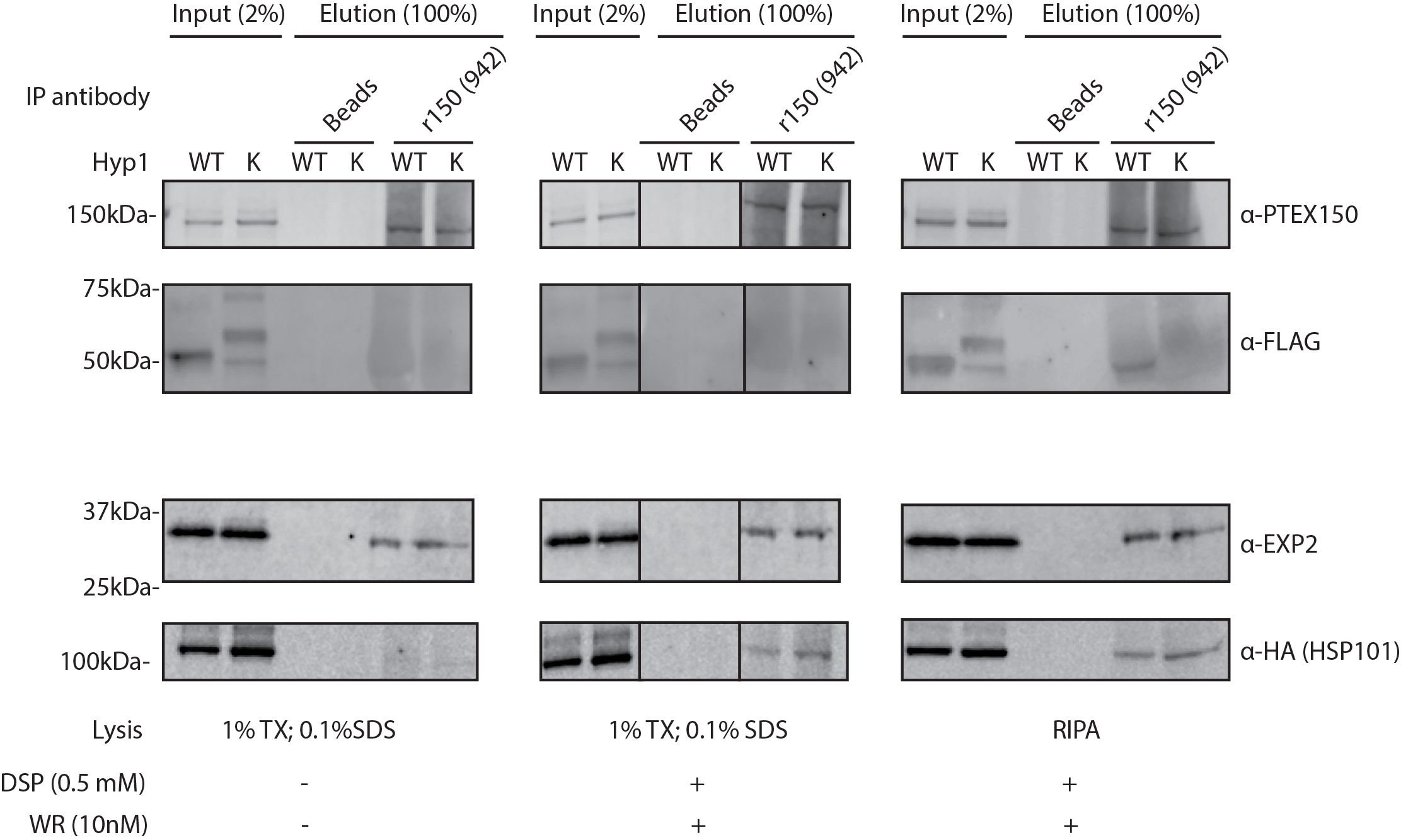

Supplement: S6 Fig — PTEX150 was immunoprecipitated from HSP101-HAglms parasites expressing WT or P5 Lys Hyp1-Nluc-mDHFR-3xFLAG reporters using polyclonal anti-PTEX150 (r942; against the C-terminal region of PTEX150). The parasites were either lysed with 1% TX-100; 0.1% SDS buffer or RIPA buffer or were crosslinked +/- 0.5 mM DSP and input (2%) and eluates (100%) were fractionated by SDS-PAGE. Western blots indicate that the WT Hyp1-Nluc-mDHFR-3xFLAG reporter interacts with PTEX150 as part of the PTEX complex with EXP2 and HSP101 but not with the ER-trapped P5 Lys Hyp1-Nluc-mDHFR-3xFLAG reporter. The assay was repeated twice. (TIF) [file ppat.1009977.s006.tif]
